# Dietary Omega-3 Intake, Supplement Use and Omega-3 Status in Elite (Tier 3) Male Gaelic Football Players: A Cross-Sectional Study

**DOI:** 10.3390/nu18193261

**Published:** 2026-10-02

**Authors:** Mark Flanagan, Lisa Ryan

**Affiliations:** 1 School of Biological Sciences, Institute for Global Food Security, Queen’s University Belfast, Belfast BT9 5DL, UK; mflanagan24@qub.ac.uk; 2 School of Health, Sport Science and Nutrition, Atlantic Technological University, H91 T8NW Galway, Ireland

**Keywords:** omega-3 polyunsaturated fatty acids, Omega-3 Index, Gaelic football, elite athletes, dietary intake, sports nutrition

## Abstract

**Background/Objectives**: Omega-3 polyunsaturated fatty acids *(n*-3PUFA) are recognised for their roles in cardiovascular and brain health, with emerging evidence suggesting potential benefits for athletic performance and recovery. However, dietary intake and biological status of *n*-3PUFA remain poorly understood in athletes from intermittent team sports in Ireland, such as Gaelic football players. Through combining self-reported intake and biological assessment, this study provides, to our knowledge, the first comprehensive characterisation of *n*-3PUFA intake, supplementation practices, barriers to intake and biological status in this population. **Methods**: Players from nine intercounty Gaelic football teams (*n* = 76, 24.7 ± 3.2 years) completed a validated food frequency questionnaire to assess dietary *n*-3PUFA intake. A subgroup of participants (*n* = 20, 25.6 ± 3.5 years, 88.5 ± 5.7 kg) completed dry blood spot sampling to determine their Omega-3 Index (O3i). **Results**: A total of 63% (*n* = 48) of participants met or exceeded the European Food Safety Authority Adequate Intake (AI) of 250–500 mg/day for EPA+DHA. Supplement users were significantly more likely to meet or exceed the AI compared with non-users (89.7% vs. 35.1%; *p* < 0.001). In the subgroup assessed, the mean O3i was 6.06 ± 0.93%, with no participant achieving an O3i of ≥8%. Dietary intake was not significantly correlated with O3i (ρ = −0.09, 95% CI [−0.51, 0.37]), however, this analysis was likely underpowered due to the small subsample assessed. Barriers to fish consumption included sensory factors (29%), cooking ability (23%) and a lack of knowledge on *n*-3PUFA (20%). **Conclusions**: The subsample assessed (*n* = 20) had an O3i below the cardioprotective threshold of 8%, however, implications for performance or recovery remain unclear. Given the cross-sectional design and small biomarker sample, intake-status findings should be interpreted as descriptive. Targeted interventions addressing both intake and supplementation practices may be needed to improve *n*-3PUFA intake and status in this population.

## 1. Introduction

Gaelic football is a high-intensity, intermittent team sport, requiring players to be proficient in a multitude of physical, technical, tactical, and mental qualities [1,2,3,4]. Despite its amateur status, players can invest upwards of 30 h per week in training, competition and recovery [1,5]. Gaelic football, a sport indigenous to Ireland and governed by The Gaelic Athletic Association (GAA), is played at two main levels of competition, club and intercounty. At the club level, players represent a local club team in competitions within their county. At the intercounty level, players are selected from club teams to play for their county against other counties, representing the sport’s elite level. Due to the combination of contact, high-speed movement, and sudden changes in direction, Gaelic football exhibits a high injury rate relative to other team sports (9.2/1000 h) that is significantly higher during match play than training (49.8/1000 h versus 3.9/1000 h) [6,7,8,9,10]. With a mean time-loss of 26 days and a condensed competitive season involving repeated training and competition within an acute recovery window (<72 h), injury represents a major burden in this sport [10]. Although nutrition is a recognised pillar in athlete health, performance and recovery, poor nutritional knowledge and practices have been reported among Gaelic football players [11,12,13,14].

Omega-3 polyunsaturated fatty acids (*n*-3PUFA) are a group of fatty acids essential for human health and must therefore be obtained from diet [15]. Of relevance to athletic performance are eicosapentaenoic acid (EPA; 20:5*n*-3) and docosahexaenoic acid (DHA; 22:6*n*-3), found predominantly in oily fish (e.g., salmon, mackerel, anchovies) and other marine sources (e.g., algae, krill) [16]. While EPA and DHA can be synthesised endogenously from plant-based sources of α-linolenic acid (ALA; 18:3*n*-3), this shorter chain FA is characterised by its low conversion rates (<3% in males) and is further reduced where an individual’s dietary intake favours that of a westernised diet, characterised by a high Omega-6 polyunsaturated fatty acid (*n*-6PUFA) intake relative to *n*-3PUFA [17,18,19]. Therefore, the direct dietary intake of EPA and DHA from seafood or marine-derived supplements is generally recommended [20].

A growing body of sports nutrition research has explored the potential role of *n*-3PUFA in athlete health, performance and recovery [16,21,22,23]. Observational evidence has linked an improved *n*-3PUFA status with neuroprotection in American football players and accelerated recovery from sports-related concussion in adolescents [24,25,26]. Research within team sport and endurance athletes demonstrates that *n*-3PUFA supplementation can improve blood lipid profiles in athletes and reduce cardiovascular strain during submaximal exercise, albeit with limited evidence of translation to performance outcomes [27,28,29]. Anti-inflammatory and membrane stabilising properties further support a role for *n*-3PUFA in supporting post-exercise recovery [30,31,32,33,34]. Furthermore, when consumed prophylactically prior to immobilisation, *n*-3PUFA supplementation has been shown to attenuate disuse-related muscle atrophy in female athletes [35]. While inflammation, recovery, injury risk, concussion and performance are discussed throughout this manuscript in relation to *n*-3PUFA status, these outcomes were not directly measured in the present study. Nonetheless, this growing body of evidence collectively underscores a need for sport-specific *n*-3PUFA recommendations.

The Omega-3 Index (O3i), defined as the combined EPA and DHA content of erythrocyte membranes, expressed as a percentage of total fatty acids, is a validated biomarker of long-chain *n*-3PUFA status over the preceding 120 days and is influenced by factors including sex, physical activity status, genetics, body mass and baseline *n*-3PUFA intake [15,36,37]. The O3i has previously been identified as a modifiable risk factor for cardiovascular disease (CVD), with risk zones being established as low (≥8%), intermediate (4–8%) and high (<4%) [15]. Using these thresholds, the general Irish population has moved from high risk <4% in 2016 to intermediate risk (4.46%) in 2024 [38]. While no athlete-specific O3i cutoffs currently exist, evidence indicates that most team sport athletes fall into the intermediate to high-risk categories with a very small minority achieving an O3i ≥8% [25,39,40,41,42,43,44,45,46]. Consequently, athletes with a low O3i may be placed at increased cardiovascular risk, with emerging evidence suggesting possible implications for performance and recovery [24,25,47,48,49,50].

Methodological heterogeneity across the current evidence base has resulted in a lack of clearly defined *n*-3PUFA recommendations for athletes such that nutrition practitioners typically follow current dietary guidelines set out for the general population or provide advice on a case-by-case basis, often relying on recommendations that still lack a solid evidence base [51,52,53]. While the European Food Safety Authority (EFSA) recommends a combined daily adequate intake (AI) of 250–500 mg EPA and DHA, equating to 1–2 servings of fish per week (one of which oily), the extent to which these recommendations are firstly met, and secondly, are effective in improving *n*-3PUFA status in Gaelic football players is still largely unknown.

This study therefore aimed, primarily, to assess the dietary intake and supplementation practices of *n*-3PUFA in elite male Gaelic footballers, including fish and seafood consumption, and EPA, DHA, and ALA intake relative to current dietary recommendations. Where dietary recommendations were not met, we aimed to identify the most prevalent barriers to *n*-3PUFA consumption. As a secondary objective, this study sought to measure the biological status of *n*-3PUFA in a subgroup of participants and compare this against self-reported dietary intake of *n*-3PUFA. We hypothesised that most players would fail to meet general *n*-3PUFA dietary guidelines and players in the subgroup would fail to have an O3i of ≥8%. Exploratory analyses examined the association between fish consumption frequency and EPA+DHA intake in the full cohort, and the relationships between self-reported intake and corresponding erythrocyte fatty acid composition in the subgroup, which included whether *n*-3PUFA intake predicted *n*-3PUFA status, measured using the O3i. This research aims to inform future interventions targeting an improved *n*-3PUFA intake, supplementation practices and status in this population. To our knowledge, this is the first study to comprehensively characterise *n*-3PUFA dietary intake, supplementation practices, associated barriers, and biological status within an elite intercounty Gaelic football cohort.

## 2. Materials and Methods

### 2.1. Experimental Design

This study employed a cross-sectional, observational design to assess the relationship between dietary intake and biological status of *n*-3PUFA in elite (tier 3) male Gaelic football players. Players were classified as “tier 3” elite athletes (i.e., competing at a national level) based on the characteristics outlined in the published participant classification framework [54]. Players participated in the study during their pre-season period. As this is, to the best of our knowledge, the first study to characterise *n*-3PUFA intake, supplementation and biological status in this population, no prior effect size estimates were available to inform a formal a priori sample size calculation which is consistent with comparable studies in this area [45]. After providing informed consent, all participants (*n* = 76) completed a brief, validated *n*-3PUFA FFQ online. Of the participants who completed the FFQ, a smaller subsample was selected from a single team via convenience sampling to provide a finger-prick dry blood spot (DBS) sample on site at a training facility local to the team. Twenty-two participants were identified for DBS sampling and completed the FFQ. Of these, two subsequently did not complete DBS sampling, resulting in a final DBS subsample of 20. Selection was determined by the availability of players from this team to participate in DBS sampling on the day of testing, as well as practical constraints related to limited DBS kit availability. As DBS was conducted at a single team’s training facility due to kit and logistical constraints, participants from the remaining eight teams did not provide a DBS sample.

### 2.2. Participants

Participants were recruited using purposive sampling by contacting nutrition practitioners currently working within the GAA and inviting their teams to participate in this study. Eligibility criteria included being a current member of a male, senior intercounty Gaelic football team and being over the age of 18 years. While formally amateur in status, the target participants represent a select group of players within the GAA who compete at a national level and are frequently required to undertake multiple trainings and games within reduced recovery periods (<72 h). In comparison to their sub-elite club-level counterparts, intercounty athletes compete in a 70 min match comprising two 35 min halves [2]. Due to the high speed and contact nature of the sport, athletes are placed at an increased risk of injury [10]. All procedures received approval from the local research ethics committee (ID: TPREC_2026_MF) in accordance with the Declaration of Helsinki. Written and verbal consent was obtained from all players prior to participation.

### 2.3. Food Frequency Questionnaire

The previously validated *n*-3PUFA FFQ used in this study (Supplementary File S1: [Online Food Frequency Questionnaire]) was administered to participants online using Microsoft Forms (Microsoft Corporation, Redmond, WA, USA) during the pre-season period, taking approximately 5 min to complete [55,56]. While this FFQ was originally validated in a clinical and subsequently a general population, it has not been formally validated in athletes, though it has been previously applied in athletic cohorts of a similar nature [40,42,43,45]. The core 21 questions which made up the basis of the FFQ aimed to assess *n*-3PUFA intake in the previous 6 months. Intakes of ALA, EPA and DHA were reported from a variety of different sources, including seafood, fish oil supplements and *n*-3PUFA-fortified foods, as well as plant-based sources of *n*-3PUFA such as flaxseed, chia seed, walnut, flaxseed oil, rapeseed oil and vegetable margarine. Four additional questions were added to the original FFQ. The first question assessed perceived barriers to achieving *n*-3PUFA recommendations when fish and/or seafood had not been consumed at least twice per week in the previous 6 months. The second identified where the player sourced their *n*-3PUFA information. Both questions were based on those of previous literature [45,57]. The threshold of twice per week reflects the upper range of the EFSA fish consumption recommendation (1–2 servings per week), capturing participants not achieving the full recommended range rather than those falling below the minimum. Participants could select multiple barriers from the response options provided. Free text “other” responses were reviewed and, where applicable, manually assigned to existing or additional thematic categories based on participant comments. The remaining questions were developed by the research team and consisted primarily of closed-ended multiple response items, with opportunities for participants to provide additional free-text responses where applicable. The questions aimed to address previously identified gaps within the literature relating to *n*-3PUFA supplement practices. These questions aimed to better understand why participants did or did not supplement with an *n*-3PUFA supplement in the previous six months. Where a participant supplemented with an *n*-3PUFA or fish oil supplement in the previous six months, the supplement dose, frequency, brand and type were recorded. Daily EPA and DHA intake from supplements was calculated by multiplying the EPA and DHA content per dose unit, which was based on manufacturer label values, by reported dose and frequency of consumption. Reported frequency categories reflect participants’ typical use of the previous six-month recall period and were converted to daily equivalents. Where a participant reported using more than one product, a midpoint estimate of the respective product’s label content values was used.

Intakes of *n*-3PUFA, specifically ALA, EPA, and DHA, were calculated based on the types of foods consumed, the reported portion sizes, the frequency of consumption, and the average *n*-3PUFA content of each food item. The *n*-3PUFA content was obtained from published food composition tables [58,59]. Where multiple species of fish were consumed, the total amount of *n*-3PUFA consumed from each species was divided by the number of species consumed, a method which has been previously validated in quantifying *n*-3PUFA intake using an FFQ [56]. Average daily *n*-3PUFA intake was calculated and used to determine if participants met EFSA guidelines of an AI of 250–500 mg/day EPA and DHA and an AI of 1.6 g/day ALA set out by the Institute of Medicine (IOM) [60,61]. For the purposes of classification, participants consuming 250–500 mg/day EPA and DHA were considered to have met the EFSA recommendations, with those consuming <250 mg/day considered below the AI and those consuming >500 mg/day considered to exceed the upper end of the AI range. For the purposes of classification, participants were considered to meet the EFSA fish consumption recommendation if they reported once per week, the lower bound of the 1–2 servings per week range, consistent with the classification approach used for EPA+DHA intake relative to the EFSA AI.

### 2.4. n-3PUFA Status

After completion of the FFQ, a smaller subgroup of participants (*n* = 20) provided a DBS sample as a secondary measure in this study. Blood samples were collected by a registered Sport and Exercise Nutrition (SENr) practitioner from the lateral side of the index finger of the participant’s non-dominant hand. Following removal of the initial blood drop, capillary whole blood was collected onto a DBS collection card supplied by OmegaQuant (OmegaQuant LLC, Sioux Falls, SD, USA), through its European partner laboratory at the University of Stirling (Stirling, UK). The collection card was pre-treated with a proprietary antioxidant to prevent the oxidative loss of PUFAs. Once a sufficient blood sample was provided, the DBS sample was allowed to dry for 15 min at room temperature before being sealed and sent the following day via post to the laboratory for analysis. Antioxidant-treated DBS cards have been shown to preserve PUFA composition at ambient temperature during transport and storage. Analysis was conducted following OmegaQuant’s standardised, validated analytical procedures. Laboratory personnel analysing the DBS samples had no access to participants’ FFQ data. At the laboratory, fatty acids were extracted from one punch (10 uL) of blood from the DBS card and quantified using gas chromatography, with identification and calibration performed against a certified fatty acid standard. Individual fatty acid values were calculated on a whole blood basis, with coefficients of variation <6%. The O3i was defined as erythrocyte EPA+DHA and derived from the whole blood EPA+DHA value using a validated regression equation (r = 0.97; CV < 5%). For further detail regarding this DBS analytical methodology, please refer to previous work in this area [37,41].

### 2.5. Statistical Analysis

The analyses corresponding to the study’s primary (dietary intake, supplementation practices, and barriers to consumption) and secondary (descriptive comparison of dietary intake with O3i) objectives were pre-specified. All remaining analyses, including correlation, regression and subgroup comparisons, were exploratory and hypothesis-generating. Statistical analyses were conducted using IBM SPSS Statistics (version 30; IBM Corp., Armonk NY, USA). Data were assessed for normality using the Shapiro–Wilk test. Normally distributed continuous variables are reported as mean ± standard deviation, whereas non-normally distributed variables are presented as median (interquartile range). Categorical variables are expressed as frequencies and percentages. Wilcoxon signed-rank tests were used to compare median ALA intake with the IOM recommended intake of 1.6 g/day for adult males. Differences in EPA and DHA intake between supplement users and non-users were assessed using the Mann–Whitney U test, while group differences in the proportion of participants meeting or exceeding the EFSA AI (≥250 mg/day), comparing supplement users with non-users, were evaluated using the Chi-square test of independence. All expected cell counts for the chi-square analysis exceeded 5, satisfying the assumptions of the test. The 95% CI for the proportion meeting the EFSA AI was calculated using the Clopper-Pearson (exact) method. Age was compared between the DBS subgroup and remaining cohort using Welch’s *t*-test. Differences in EPA, DHA, EPA+DHA, and ALA intake, and in fish consumption frequency between the DBS subgroup and remaining cohort were also assessed using the Mann–Whitney U test, while group differences in the proportion meeting or exceeding the EFSA AI and reporting supplement use between these groups were evaluated using the Chi-square test of independence. Differences in fish-derived EPA+DHA intake between supplement users and non-users were also assessed using the Mann–Whitney U test. Associations between dietary *n*-3PUFA intake (g/day), fish consumption frequency (servings/week) and erythrocyte fatty acid composition (%*w*/*w*) were examined using Spearman’s rank correlation coefficients (ρ). Simple linear regression analysis was conducted to assess whether total dietary EPA and DHA intake predicted O3i. Model assumptions for the linear regression were assessed, including normality of residuals (Shapiro–Wilk test) and homoscedasticity (visual inspection of standardised plots), alongside Cook’s distance to identify influential observations. Sensitivity analyses were conducted for both the regression and Spearman’s correlations by re-running each with the highest-intake observations excluded. Statistical significance was set at *p* < 0.05 for all analyses.

## 3. Results

### 3.1. Participant Data

A total of 76 players from nine different senior male intercounty football teams with a mean age of 24.7 ± 3.2 years met the inclusion criteria and successfully completed the brief validated *n*-3PUFA FFQ online. No participants were excluded as all met the inclusion criteria required to complete the FFQ, and no responses were incomplete or missing. Of the 76 players who successfully completed the online FFQ, 22 were selected via convenience sampling to provide a DBS sample for fatty acid analysis. Two participants did not continue with DBS sampling, which left a final subgroup of *n* = 20 (25.6 ± 3.5 years, 88.5 ± 5.7 kg).

### 3.2. Dietary Intake

**Figure 1:**
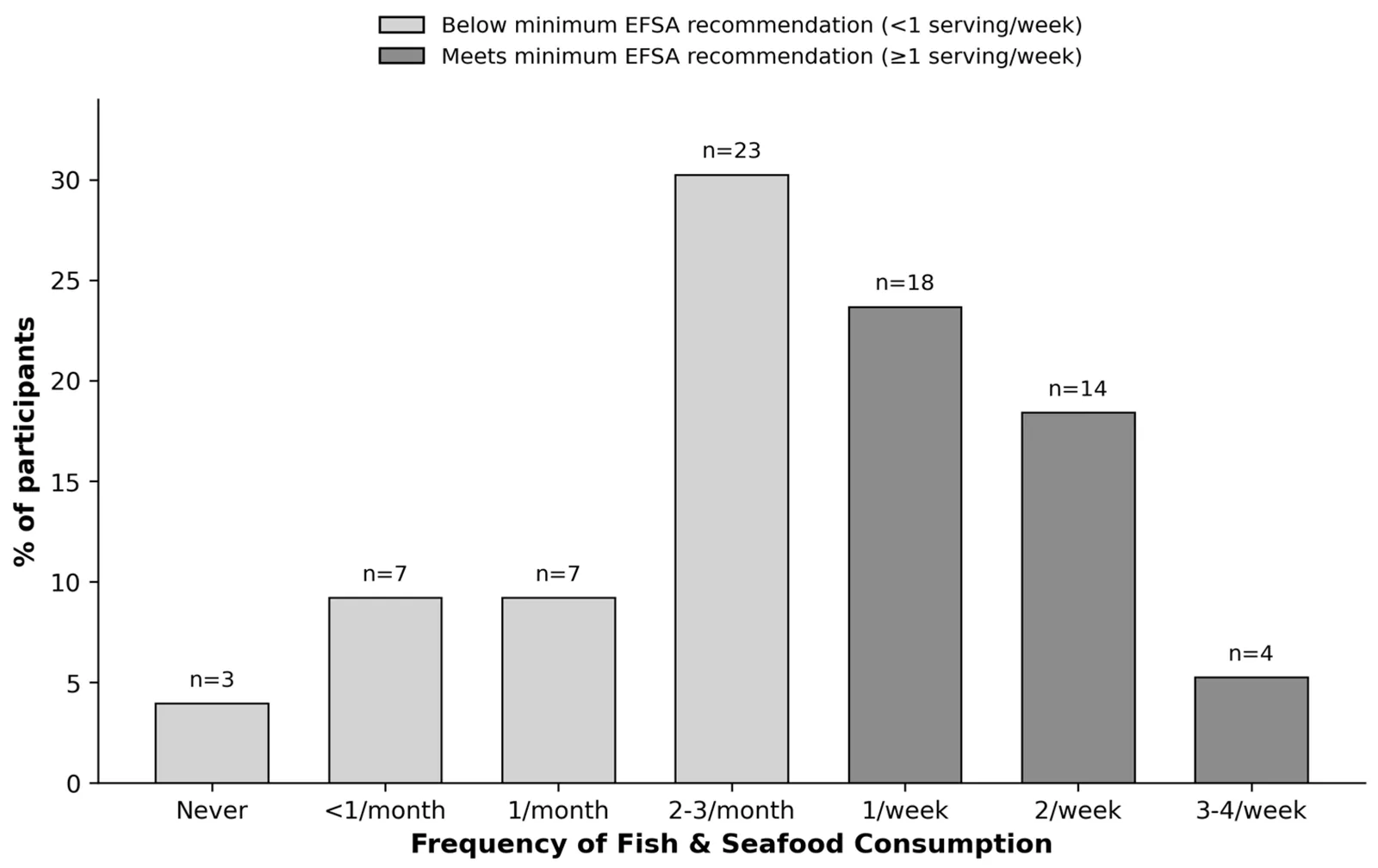
Distribution of fish and seafood consumption frequency over the previous 6 months among participants (*n* = 76). Bars represent the percentage of participants in each intake category, with numbers indicating sample size per category. Bars are shaded to indicate categories meeting (≥1 serving/week, dark grey) versus not meeting (light grey) the European Food Safety Authority (EFSA) recommendation.

**Figure 2:**
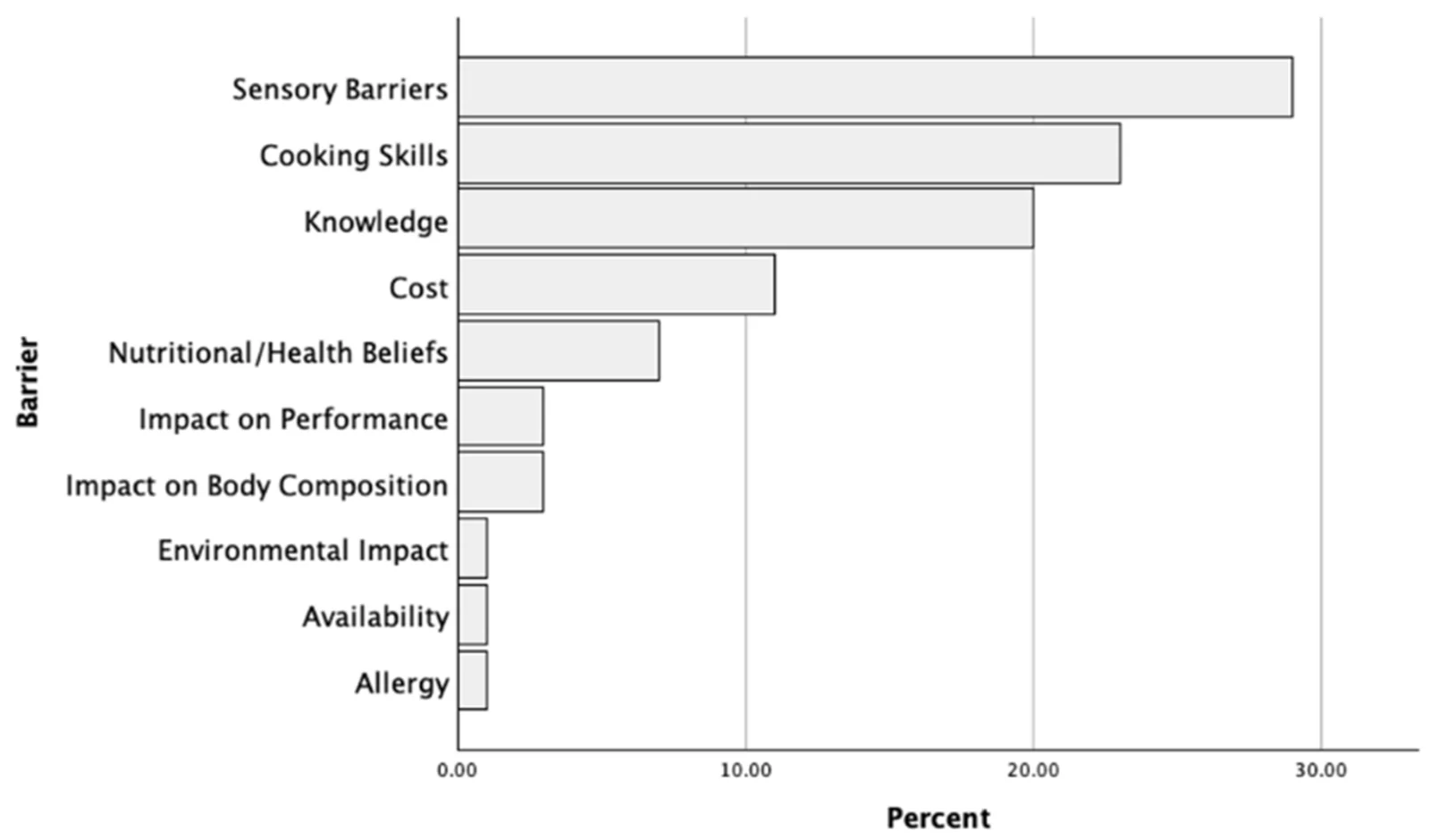
Prevalence of self-reported barriers to fish and seafood consumption among elite male intercounty Gaelic football players who had not consumed fish at least twice per week in the previous six months (*n* = 40). Values represent each barrier’s percentage share of total selections across all respondents (participants could select multiple barriers).

Among the 76 participants who successfully completed the online self-reported *n*-3PUFA FFQ, 47.4% (*n* = 36) reported fish intakes meeting the minimum EFSA recommendation of ≥1 serving per week (Figure 1). In the present study, salmon was the most frequently consumed fish (*n* = 57), followed by cod (*n* = 47), tuna (*n* = 32), hake (*n* = 25), haddock (*n* = 23), sole (*n* = 5), monkfish and sea bass (both *n* = 3). Among oily fish, a key dietary source of *n*-3PUFA, salmon was the dominant contributor, with limited players reporting consumption of mackerel (*n* = 11), trout (*n* = 7), sardines (*n* = 2), and herring (*n* = 1). Shrimps/prawns/scampi were the most consumed shellfish (*n* = 33), followed by mussels (*n* = 15), crab and octopus/calamari (both *n* = 10), lobster (*n* = 6), scallops and oysters (both *n* = 4). As part of the FFQ, barriers were assessed among participants who had not consumed fish at least twice per week in the previous six months. Sensory factors (29%), cooking skills (23%) and a lack of knowledge on the potential benefits of *n*-3PUFA (20%) were the most prevalent barriers reported. The remaining barriers to fish consumption included cost (11%), health and nutritional beliefs (7%), perceived negative impact on performance (3%) and body composition (3%), allergy (1%), environmental impact (1%) and lack of availability (1%) (Figure 2). A qualified nutritionist/dietician was the most frequently cited source of *n*-3PUFA information (*n* = 44), followed by internet/social media (*n* = 30), other members of sports staff (*n* = 8), another athlete (*n* = 5), and family/friend (*n* = 4). No athletes reported sourcing information from research papers or scientific journals.

Median intake of EPA and DHA was 0.41 g/day (IQR: 0.82 g; range: 0.01–7.91 g/day; *n* = 76). Categorisation of total daily EPA and DHA intake showed that 28 participants (36.8%) consumed <250 mg/day, 13 participants (17.1%) consumed 250–500 mg/day, and 35 participants (46.1%) consumed >500 mg/day. Overall, 63.2% (*n* = 48, 95% CI [51.3%, 73.9%]) met or exceeded the EFSA AI range of 250–500 mg/day EPA and DHA. Median daily ALA intake was 0.42 g/day (IQR: 1.23 g/day), which was significantly lower than the recommended 1.6 g/day (Z = 4.978, *p* < 0.001), with only 12 participants (15.8%) meeting current ALA recommendations.

*n*-3PUFA supplement use was reported by 39 participants (51%) across nine different brands, with the most reported products containing 15.7–68.75 mg EPA and 11.1–25 mg DHA per 100 mg of product (label values). Frequency of consumption ranged from 2 to 3 times per month to once daily. Calculated EPA+DHA intake from supplements, derived from reported brand, dose and frequency, ranged from 60 mg to 7500 mg across the sample. The primary reasons for supplementation included enhanced recovery (24%), improved cognitive function (22%), general health (22%) and reduced inflammation (18%). Where an *n*-3PUFA supplement was not used by a participant in the previous 6 months, a lack of knowledge was the most cited reason (65%), followed by cost (15%). Supplement users (*n* = 39) had significantly higher EPA+DHA intakes than non-supplement users (*n* = 37) (mean rank = 48.04 vs. 26.38; U = 283.50, Z = −4.332, r = 0.50, *p* < 0.001; Table 1). This pattern was consistent for EPA and DHA individually (both *p* < 0.001; Table 1) but not for ALA intake, which did not differ significantly between groups (*p* = 0.513). Furthermore, supplement use was strongly associated with meeting or exceeding EFSA AI recommendations for EPA+DHA (χ^2^(1) = 24.33, *p* < 0.001; OR = 16.15, 95% CI [4.70, 55.55]), with 89.7% (*n* = 35) of supplement users meeting or exceeding 250 mg/day compared with 35.1% (*n* = 13) of non-users. Fish-derived EPA and DHA intake did not differ significantly between supplement users and non-users (Table 1), indicating that higher total intake among supplement users was not attributable to differences in dietary fish consumption. Among supplement users, supplement-derived intake accounted for the majority of total EPA+DHA intake (Table 1). Conversely, 24 of 37 non-users (64.9%) consumed <250 mg/day EPA+DHA, compared with 4 of 39 supplement users (10.3%). The DBS subgroup reported significantly higher dietary EPA (U = 808.0, r = 0.34, *p* = 0.004), DHA (U = 755.0, r = 0.26, *p* = 0.022) and EPA+DHA intake (U = 803.0, r = 0.33, *p* = 0.004), with a small effect size for the non-significant difference in ALA intake (U = 625.0, r = 0.09, *p* = 0.447). A significantly greater proportion met the EFSA AI (OR = 4.57, 95% CI [1.20, 17.38]) and reported supplement use (OR = 4.00, 95% CI [1.28, 12.53]) compared with the remaining cohort (Table 2). Fish consumption frequency did not differ significantly between the subgroup and remaining cohort (median 1.0 vs. 0.6 servings/week equivalent; U = 680.5, *p* = 0.147), indicating that the subgroup’s higher calculated EPA+DHA intake was attributable to differences in supplementation practices rather than dietary fish consumption. Age did not differ significantly between the DBS subgroup and the remaining cohort (25.6 ± 3.5 vs. 24.4 ± 3.1 years; t(30.3) = 1.37, *p* = 0.182). No significant difference was observed for ALA intake between the groups.

### 3.3. Biological Status

The mean O3i of the subsample of participants assessed (*n* = 20) was 6.06 ± 0.93%, with values ranging from 3.64 to 7.31%. Whole blood EPA, DHA and ALA accounted for 1.18 ± 0.37%, 2.98 ± 0.58%, and 0.41 ± 0.12% of total fatty acids, respectively. As O3i is derived from whole blood EPA+DHA through a validated regression equation representing an erythrocyte-equivalent value, rather than a direct arithmetic sum of individual whole blood fatty acids, these values are not directly additive. One participant was classified as high risk for CVD with an O3i of 3.64% while no participant had an O3i of ≥8%.

**Figure 3:**
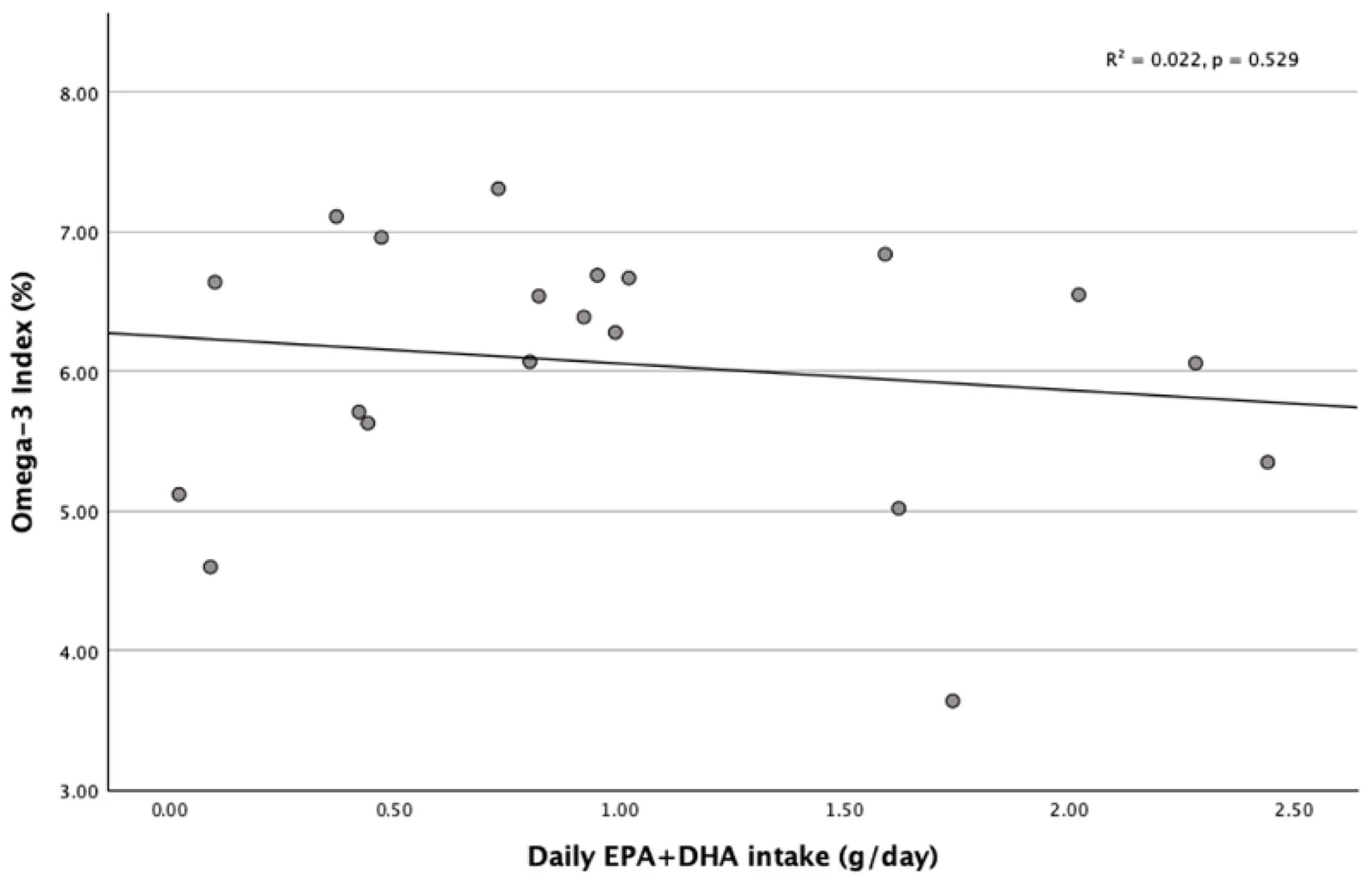
Scatter plot showing the relationship between daily EPA and DHA intake (g/day) and Omega-3 Index (%) in the DBS subsample (*n* = 20). The regression line represents simple linear regression (β = −0.150, *p* = 0.529).

In the subset of participants who provided DBS samples (*n* = 20), mean fish consumption frequency was 1.34 ± 1.08 servings per week (range: 0–3.5 servings/week) and mean intake of EPA+DHA was 0.99 ± 0.73 g/day, with 75% (*n* = 15) of participants in the subsample reporting supplement use in the previous 6 months. Spearman’s rank correlations were used to examine associations between dietary *n*-3PUFA intake, fish consumption frequency and erythrocyte fatty acid composition. Although there was no significant association between fish consumption frequency and O3i (ρ = 0.158, 95% CI [−0.31, 0.56], *p* = 0.507; *n* = 20), the wide confidence interval reflects the limited precision of this estimate at this sample size rather than confirmed evidence of no relationship. As shown in Table 3, no significant correlations were observed between dietary intake of *n*-3PUFA and the corresponding blood fatty acid measures, including O3i (*p* > 0.05 for all). However, the 95% confidence intervals for all correlation coefficients were wide and spanned zero. Simple linear regression was conducted to examine whether total dietary intake of EPA and DHA predicted O3i. The model was not statistically significant (F(1,18) = 0.413, *p* = 0.529) and explained 2.2% of the variance in O3i (R^2^ = 0.022). Dietary intake of EPA and DHA was not a significant predictor of O3i (B = −0.192, SE = 0.298, 95% CI [−0.819, 0.435], β = −0.150, t(18) = −0.643, *p* = 0.529; Figure 3). However, the wide confidence interval around this estimate reflects limited power at this sample size to detect a true effect. Model diagnostics indicated no evidence of heteroscedasticity on visual inspection of standardised residual plots, and residuals did not significantly deviate from normality (Shapiro–Wilk *p* = 0.070). Two observations exceeded the Cook’s distance threshold, though a sensitivity analysis excluding these produced a comparable result (*p* = 0.480).

## 4. Discussion

The principal findings from this present study were threefold. First, our hypothesis that most players would fail to meet general *n*-3PUFA dietary guidelines was supported for fish consumption frequency (1–2 servings/week) and ALA intake (1.6 g/day) but not for EPA+DHA intake, as 63.2% of participants met or exceeded the EFSA AI (250–500 mg/day). Sensory barriers, cooking skills and limited knowledge were cited as the most common barriers to fish consumption. Second, although supplement use was associated with significantly higher EPA+DHA intakes, this did not translate into participants reaching a cardioprotective O3i of ≥8%. Given the cross-sectional design, small subsample and small number of non-supplement users within this subgroup (*n* = 5), this observation should not be interpreted as evidence regarding the efficacy of supplementation in improving *n*-3PUFA status. Third, no significant association was observed between self-reported dietary intake of *n*-3PUFA and O3i in this subsample (*n* = 20). However, a greater sample size is needed to determine whether a true association exists between self-reported dietary intake of *n*-3PUFA and O3i in this population.

### 4.1. n-3PUFA Dietary Intake and Supplement Practices

A notable observation from this study is the distinction between meeting the EFSA fish consumption frequency (1–2 servings per week) and meeting the EFSA EPA+DHA intake recommendation (250–500 mg/day), two related but separate targets. While 47.4% of participants met the minimum fish consumption frequency, 63.2% met or exceeded the EPA+DHA intake recommendation. This apparent discrepancy likely reflects two factors. Firstly, the EPA+DHA content of fish varies substantially between species and portion sizes, such that meeting a frequency-based target does not guarantee an equivalent intake of EPA+DHA, particularly given that aside from salmon, oily fish consumption in this cohort was minimal. Secondly, supplementation contributed substantially to meeting EPA+DHA recommendations, independent of fish consumption, accounting for 80.6% of total EPA+DHA intake among supplement users (Table 1). Taken together, these findings suggest that meeting EPA+DHA intake recommendations was driven more by supplementation and, to a lesser extent, fish species selection than by adherence to fish consumption frequency guidelines alone.

Previous research has demonstrated that dietary consumption of *n*-3PUFA in athletes is often inadequate in meeting general dietary recommendations [40,42,43,45,46]. However, we found that while the majority of participants failed to meet EFSA fish consumption guidelines of 1–2 servings per week, the median reported intake of EPA and DHA was 0.41 g/day, which is greater than that of previous research in male National Collegiate Athletic Association NCAA athletes (159.8 mg/day) and that of the general Irish population aged 18–35 (201.8 mg/day) but significantly less than that of male International Rugby 7s players (1908 mg/day) [42,43,62]. Given the high prevalence of supplement use in this population (51%), our research is consistent with previous literature whereby a greater proportion of supplement users met dietary recommendations for EPA and DHA compared with non-users [43]. Supplement use in the present study was considerably higher than reported in a recent large-scale Australian athlete cohort (19%), which may reflect differences in nutritional support structures, sport type or supplementation practices between cohorts [63].

The present findings highlight substantial inter-individual variability in *n*-3PUFA intake within this cohort, with reported intakes ranging from <50 mg/day to values exceeding that of the EFSA safe reference intake of 5 g/day [64]. Prior literature suggests very low EPA and DHA intakes (<100 mg/day) fall well below established recommendations and may compromise membrane fatty acid composition and anti-inflammatory capacity, with potential implications for recovery and susceptibility to exercise-induced inflammation and injury [47,48], however, these outcomes were not directly assessed in the present study. Conversely, while empirical data on chronic intakes above 5 g/day *n*-3PUFA remain limited, theoretical concerns exist regarding altered platelet function, bleeding risk and blunting of training adaptation. However, current clinical evidence to support these effects is limited [16,65,66].

The primary motivations for supplement use in the present study included enhanced recovery (23%), cognitive function (22%), and general health (22%). While supplementation was associated with a higher *n*-3PUFA intake, no participant reporting supplement use achieved an O3i of ≥8% in the subsample assessed. This pattern is consistent with a broader distinction between dietary intake and biological incorporation, which suggests that factors beyond total intake may influence *n*-3PUFA status. Given the cross-sectional design and small DBS subsample, these observations should be interpreted as descriptive rather than evidence regarding the efficacy of supplementation or the broader relationship between intake and biological incorporation. The considerable variability seen in supplementation practices within this cohort may be one contributing factor, though this was not directly tested. Supplement use was associated with significantly higher intakes of both EPA and DHA individually, though the magnitude of increase was greater for EPA. Supplement-derived EPA (median 0.50 g/day) was approximately 2.5-fold higher than supplement-derived DHA (0.20 g/day: Table 1), consistent with the EPA-dominant formulation of many commercially available fish oil products. A further explanation relates to the differing timeframes captured by the FFQ, which assesses habitual intake over the preceding six months, whereas the O3i reflects a shorter period of approximately 120 days. As dietary and supplement practices are not necessarily stable across six months, this temporal mismatch may limit the interpretability of the observed correlations. However, given the wide confidence intervals observed around the intake-status correlations in this subsample, this study was likely underpowered to detect a true association such that these observations should be confirmed in a larger sample. The absence of an association between self-reported intake and O3i in this subsample may reflect several factors beyond the reliability of self-reported intake itself, including measurement error inherent to FFQ-based estimation, inconsistent or irregular supplement use, variation in supplement composition and the temporal mismatch between dietary and biomarker assessment windows, rather than indicating that dietary intake is unrelated to biological status.

Self-reported *n*-3PUFA dietary intake reflected each player’s habitual intake over the 6 months preceding pre-season, including a period where players were removed from their elite environment and returned to their club. During this period, dietary intake and supplement practices may differ, however, due to the limitations of the FFQ, the extent of these changes could not fully be captured. Notably, nine different fish oil supplements were reported across the nine teams, with several players reporting use of more than one product across the 6-month period. The lack of association between intake and status could be reflected by the variability that exists within the fish oil industry in fish species, lipid forms, and discrepancies between labelled and actual EPA and DHA [67,68,69]. Collectively, this underscores the need for year-round education on high-quality, third-party batch-tested *n*-3PUFA products, as improved off-season practices may increase the likelihood of athletes entering the pre-season period with an improved *n*-3PUFA status while ensuring safe supplementation in this population [70].

### 4.2. Barriers to Consumption

An important determinant of *n*-3PUFA intake and tissue incorporation is the species of fish consumed, with oily fish providing the greatest source of dietary *n*-3PUFA [71]. In the present study, salmon (*n* = 57) was the most frequently consumed oily fish, with consumption of other oily fish species being minimal in this population. Three participants reported no consumption of any species of fish in the previous six months. These findings highlight the need to increase athlete awareness on *n*-3PUFA-rich fish species and, where fish intake is not feasible, highlight the role fish oil supplements and *n*-3PUFA-enriched foods can play as an alternative in achieving a sufficient *n*-3PUFA intake. This is particularly pertinent given the substantial decline in the EPA and DHA content of farmed salmon reported over the last decade [72,73].

Reported barriers to fish consumption included sensory factors (29%), cooking skills (23%) and a lack of knowledge on *n*-3PUFA (20%), which are similar to those reported in previous work in female Irish Olympic team sport athletes [45]. Consequently, strategies aiming to improve *n*-3PUFA intake should prioritise athlete education on the relevance of *n*-3PUFA in their health and performance, alongside provision of practical recommendations to support achieving current dietary guidelines in a realistic manner [74]. Encouragingly, most players sourced their *n*-3PUFA information from a qualified nutritionist/dietician, reflecting the value of professional nutrition support and the role of qualified practitioners in translating evidence-based recommendations in this population.

Availability of oily fish at mealtimes was also reported as a barrier in achieving dietary recommendations, therefore, in line with previous research, provision of oily fish rich in EPA and DHA during mealtimes can positively impact O3i [75]. Additionally, improving an athlete’s cooking abilities could serve as an indirect way of increasing oily fish intake and enhancing subsequent *n*-3PUFA status [76]. However, where a food-first approach is not practical, the use of high-quality fish oil supplements or *n*-3PUFA-enriched foods can serve as a viable alternative in improving *n*-3PUFA status [77,78]. Collectively, these findings provide a baseline to inform future interventions targeting an improved dietary intake of *n*-3PUFA in elite male intercounty footballers in Ireland.

### 4.3. Omega-3 Index

The mean O3i of the subsample assessed in this study (*n* = 20) was 6.06 ± 0.93%, which, while suboptimal according to CVD literature, exceeds O3i values recorded for the general population in Ireland (4.46%) [15,38,79]. In comparison to other research examining O3i in team sport athletes, participants in the present study had O3i values greater than those reported in male International Rugby 7s players (4.3 ± 0.81%), male NCAA American football players (4.48% ± 1.12%) and female Olympic athletes (5.19 ± 0.86%) [42,43,45]. In comparison with more recent, larger athletic cohorts, the mean O3i observed in the present study was notably higher than that reported in a large multi-sport Australian cohort (5.88 ± 1.40%) and a pooled analysis of 1452 athletes across 18 studies (4.43 ± 0.59%) [63,75]. This discrepancy likely reflects the substantially larger cohort of athletes available relative to the present subsample. Despite this, uncertainty remains as to the effectiveness of public health recommendations in achieving an O3i of ≥8%, particularly in athletes, whereby several different factors impact *n*-3PUFA metabolism and subsequent tissue incorporation [36,80,81]. However, given the small (*n* = 20), single-team, cross-sectional sample, these findings should be interpreted cautiously. Further research is needed to identify whether athlete-specific O3i thresholds exist, and if so, whether achieving them results in meaningful improvements in performance and/or recovery, alongside practical dietary recommendations to support their achievement.

### 4.4. Strengths and Limitations

To the best of our knowledge, this is the first cross-sectional, observational study to characterise dietary intake and the biological status of *n*-3PUFA using this combination of methods in male intercounty Gaelic football players, who represent a select elite cohort within an amateur sport. Given the small, homogenous sample, the generalisability of these findings is limited to this population and should not be extended to athletes of other sports, competitive levels, or sexes, who differ in training demands, dietary habits, *n*-3PUFA metabolism, and access to structured nutritional support. Additionally, as participants were recruited through nutrition practitioners working within the GAA, it is possible that athletes with greater existing access to nutritional support, or greater interest in nutrition, were more likely to participate. This may limit the generalisability of these findings. As players were recruited from nine teams, participants within the same team may have shared nutritional practices, supplement access or support, potentially resulting in within-team clustering of dietary intake or *n*-3PUFA status. To protect anonymity, team-level participant numbers were not recorded, such that formal assessment of this clustering could not be performed. As the DBS subsample was drawn entirely from a single team, the biomarker and intake findings from that cohort may reflect team-specific factors rather than being representative of the wider nine-team cohort. Anthropometric and sport-related characteristics were not collected in the full cohort, limiting our ability to fully characterise the sample such that future research should aim to incorporate these variables where possible. Nevertheless, combined with that of previous work, the present findings contribute to the building evidence relating to *n*-3PUFA status and intake in athletic populations. Future research should prioritise larger, multi-team samples incorporating objective biomarker assessment alongside dietary intake to determine whether the intake-status disconnect observed in this study reflects a broader pattern in this population and whether athlete-specific O3i thresholds exist.

A key strength of this study was the combined use of self-reported *n*-3PUFA intake using the FFQ alongside the objective biomarker of *n*-3PUFA derived from DBS sampling. This approach provided complementary insight across dietary and biological measures of *n*-3PUFA status across multiple time scales, however, these methods are not without their own inherent limitations. Firstly, the DBS subgroup reported a significantly higher EPA, DHA and EPA+DHA intake and supplement use compared with the remaining cohort (Table 2), reflecting non-random, convenience-based selection. The O3i from this subsample may therefore not fully represent the *n*-3PUFA status of the wider cohort. In relation to self-reported *n*-3PUFA intake, FFQs are subject to recall bias, misreporting of portion sizes, and social desirability bias, which may be exacerbated in athletic populations where dietary practices and supplement use are often irregular, periodized, or influenced by competition schedules [82,83]. This highlights a further limitation in that while this FFQ has been previously applied in athletic populations, it has not been formally validated in athletes. Furthermore, FFQs assess habitual intake over extended periods and may not adequately capture recent changes in *n*-3PUFA intake, such as short-term supplementation or acute dietary changes, both of which are prevalent in athletic populations. Similarly, reported frequency of supplement use was treated as constant over this period as the FFQ did not separately capture or adjust for irregular or inconsistent use, which may not reflect real-world supplementation patterns in a cohort of athletes. Duration of supplement use prior to testing was not captured by the FFQ, which assessed supplement use only within the preceding six-month recall period. Supplement use, dose and frequency were self-reported, and adherence was not objectively verified such that actual consumption may therefore have differed from reported use. Additionally, the estimation of *n*-3PUFA intake from FFQ data depends on food composition databases which may not account for variability in *n*-3PUFA content due to differences in fish species, food sourcing, preparation methods, time of year or supplement formulations. Consequently, absolute intake values should be interpreted with caution. Similarly, the method used to distribute *n*-3PUFA intake evenly across multiple reported fish species does not account for potential differences in the relative quantity of each species consumed, which may introduce measurement error given the substantial variability in EPA and DHA content between species. Lastly, reference ranges for *n*-3PUFA status are largely derived from general or clinical populations, and athlete-specific thresholds relevant to performance and recovery remain poorly defined. Finally, given the number of statistical comparisons conducted, particularly among the exploratory analyses, there is an increased risk of Type I error. No correction for multiple testing was applied such that exploratory findings should be interpreted as hypothesis-generating rather than confirmatory.

## 5. Conclusions

The present study demonstrated that most elite male intercounty Gaelic footballers did not achieve recommended dietary guidelines of 1–2 servings of fish per week. While 63% met or exceeded the EFSA AI of 250–500 mg/day for EPA and DHA, this was largely supplement-driven. Supplements accounted for 80.6% of total EPA+DHA intake among supplement users, with fish-derived intake being comparable to that of non-users. Within the subsample assessed, no significant associations were observed between dietary intake and erythrocyte fatty acid status, though this analysis was likely underpowered to detect a true effect. Despite reporting a higher dietary intake of EPA+DHA than the wider cohort, all players in the subsample commenced pre-season with an O3i below the CVD-threshold of 8%, suggesting that self-reported intake alone may not adequately capture *n*-3PUFA status in this population. However, as no athlete-specific O3i threshold currently exists, the implications of this finding for performance or recovery in this population remain unclear. The most cited barriers to fish consumption within this population included sensory factors, cooking ability and limited knowledge of the benefits of *n*-3PUFA. The high degree of heterogeneity seen in supplement practices highlights the need for more targeted education on safe, high-quality and effective supplementation. Given the cross-sectional design and small, single-team biomarker subsample (*n* = 20), these findings should be interpreted as descriptive rather than confirmatory in this population. Nonetheless, the current study serves as a baseline for practitioners to create targeted interventions with the aim of improving dietary intake and subsequent biological status of *n*-3PUFA in this population.

## Figures and Tables

**Table 1:** Median daily *n*-3PUFA intake in supplement users and non-supplement users.

	Supplement Users (*n* = 39)	Non-Supplement Users (*n* = 37)	*p*-Value
**Total intake, median (IQR), g/day**			
EPA	0.56 (0.71)	0.05 (0.09)	<0.001
DHA	0.32 (0.22)	0.10 (0.17)	<0.001
EPA+DHA	0.93 (0.92)	0.15 (0.92)	<0.001
ALA	0.57 (1.23)	0.27 (1.27)	0.513
**Fish-derived intake, median (IQR), g/day**			
EPA	0.04 (0.06)	0.05 (0.09)	0.483
DHA	0.07 (0.11)	0.10 (0.17)	0.451
EPA+DHA	0.11 (0.17)	0.14 (0.26)	0.489
**Supplement-derived intake, median (IQR), g/day**			
EPA	0.50 (0.68)	-	-
DHA	0.20 (0.24)	-	-
EPA+DHA	0.75 (1.00)	-	-

Values are median (interquartile range), g/day. Group comparisons were carried out using the Mann–Whitney U test.

**Table 2:** Comparison of dietary *n*-3PUFA intake and supplement use between the DBS subgroup and remaining cohort.

	DBS Subgroup (*n* = 20)	Remaining Cohort (*n* = 56)	*p*-Value
**Dietary intake, median (IQR), g/day**			
EPA (g/day)	0.57 (0.85)	0.10 (0.62)	0.004
DHA (g/day)	0.30 (0.42)	0.17 (0.32)	0.022
EPA+DHA (g/day)	0.87 (1.13)	0.28 (0.87)	0.004
ALA (g/day)	0.50 (1.16)	0.37 (1.34)	0.447
**Categorical, *n* (%)**			
Meeting or Exceeding EFSA AI	17 (85.0)	31 (55.4)	0.037
Supplement Use	15 (75.0)	24 (42.9)	0.027

Dietary intake variables were compared using the Mann–Whitney U test; categorical variables were compared using the Chi-square test of independence.

**Table 3:** Relationship between self-reported *n*-3PUFA intake and erythrocyte fatty acid status (*n* = 20).

	Whole Blood Sample (%*w*/*w*)	Daily Intake (g/day)	Spearman’s ρ (95% CI)	*p*-Value
**ALA**	0.41 ± 0.12	0.84 ± 0.98	0.17 (−0.30, 0.57)	0.468
**EPA**	1.18 ± 0.37	0.62 ± 0.49	0.19 (−0.28, 0.58)	0.431
**DHA**	2.98 ± 0.58	0.37 ± 0.30	−0.13 (−0.54, 0.33)	0.576
**O3i**	6.06 ± 0.93	0.99 ± 0.73	−0.09 (−0.51, 0.37)	0.696

Values are presented as mean ± SD. Spearman’s rho was used to assess associations between dietary intake and corresponding erythrocyte fatty acid composition. 95% confidence intervals were calculated using Fisher’s z-transformation.

## Data Availability

The data presented in this study are available on request from the corresponding author. The data are not publicly available due to participant privacy and confidentiality considerations.

## Supplementary Materials

The following supporting information can be downloaded at https://www.mdpi.com/article/10.3390/nu18193261/s1: File S1: Online Food Frequency Questionnaire.

## Abbreviations

The following abbreviations are used in this manuscript:

ALA	α-Linolenic acid
CI	Confidence interval
CVD	Cardiovascular disease
DBS	Dry blood spot
DHA	Docosahexaenoic acid
EFSA	European Food Safety Authority
EPA	Eicosapentaenoic acid
FA	Fatty acid
FFQ	Food frequency questionnaire
GAA	Gaelic Athletic Association
IQR	Interquartile range
*n*-3PUFA	Omega-3 polyunsaturated fatty acids
*n*-6PUFA	Omega-6 polyunsaturated fatty acids
O3i	Omega-3 Index
SD	Standard deviation
SEN	Sport and Exercise Nutrition
