# Supplementary material for: Dietary Omega-3 Intake, Supplement Use and Omega-3 Status in Elite (Tier 3) Male Gaelic Football Players: A Cross-Sectional Study
Source: Nutrients. 2026 Oct 2;18(19):3261. doi: 10.3390/nu18193261 (PMC13666917; doi:10.3390/nu18193261)
Supplement: Supplementary file 1 [file nutrients-18-03261-s001.zip › nutrients-4504828-supplementary.pdf]

Supplementary Material

Online Food Frequency Questionnaire

Pre-questionnaire Brief

When completing this questionnaire please be aware that we are considering a full spectrum of fish and seafood including and not limited to:
- Fresh
- Battered
- Frozen
- Soups
- Salads
- Sushi
- Smoked/Marinated
- Spreads/Pastes
- Breaded
- Tinned
- Fish Cakes
- Fish Burgers

1. How many times in the past 6 months have you consumed fish or seafood in any form? (including sushi prepared with fish or seafood) Single choice.

Never

Less than once a month

Once a month

2-3 times per month

Once a week

Twice a week

3-4 times per week

5-6 times per week

Once a day

Multiple times per day

2. **For what reasons have you not consumed fish or shellfish 2 times per week in the last 6 months?**

Sensory Barriers (smell, taste etc.)

Environmental Impact

Impact on Body Composition

Impact on Performance

Allergy

Risk of Contaminants

Not Aware of Possible Benefits

Health and Nutritional Beliefs

Cost

Cooking Skills

If selected other, please specify source below

3. **Please tick all the fish and seafood you have consumed in the previous 6 months.**

Salmon

Trout

Tuna

Herring

Hake

Haddock

Mackerel

Sole

Pike

Sardines

Carp

Cod

Anchovies

Monkfish

Caviar

Lobster

Octopus/Calamari

Scallops

Oysters

Crab

Shrimps/Prawns/Scampi

Mussels

If selected other, please specify the type of fish/seafood consumed.

4. **Each time you ate fish or seafood, how much of it did you eat?**

Less than a filet or less than 4 pieces of sushi

About 1 filet or 4-14 pieces of sushi

More than 1 filet or more than 14 pieces of sushi

5. **How many times in the past 6 months did you eat walnuts?**

Never

Less than once a month

Once a month

2-3 times per month

Once a week

Twice a week

3-4 times per week

5-6 times per week

Once a day

Multiple times per day

6. **Each time you ate walnuts, how many did you eat?**

Less than 10 nut halves (<30 grams)

10-20 nut halves (30-60 grams)

More than 20 nut halves (> 30 grams)

7. **How many times in the past 6 months have you used rapeseed/canola oil?**

Never

Less than once a month

Once a month

2-3 times per month

Once a week

Twice a week

3-4 times per week

5-6 times per week

Once a day

Multiple times per day

8. **Each time you used rapeseed/canola oil, how much of it did you use?**

Less than a Teaspoon (<5ml)

1-2 Teaspoons (5-10ml)

1 Tablespoon (15ml)

1-2 Tablespoons (15-30ml)

More than 2 Tablespoons (>30ml)

9. **How many times in the past 6 months did you eat flaxseed?**

Never

Less than once a month

Once a month

2-3 times per month

Once a week

Twice a week

3-4 times per week

5-6 times per week

Once a day

Multiple times per day

10.**Each time you ate flaxseed, how much of it did you eat?**

Less than a Teaspoon (<5g)

1-2 Teaspoons (5-10g)

1 Tablespoon (12g)

1-2 Tablespoons (12-24g)

More than 2 Tablespoons (>24g)

11.**How many times in the past 6 months did you use flaxseed oil?**

Never

Less than once a month

Once a month

2-3 times per month

Once a week

Twice a week

3-4 times per week

5-6 times per week

Once a day

Multiple times per day

12.**Each time you used flaxseed oil, how much of it did you use?**

Less than a Teaspoon (<5ml)

1-2 Teaspoons (5-10ml)

1 Tablespoon (15ml)

1-2 Tablespoons (15-30ml)

More than 2 Tablespoons (>30ml)

13. How many times in the past 6 months did you eat chia seeds?

Never

Less than once a month

Once a month

2-3 times per month

Once a week

Twice a week

3-4 times per week

5-6 times per week

Once a day

Multiple times per day

14. Each time you ate chia seeds, how much of them did you eat?

Less than a Teaspoon (<5g)

1-2 Teaspoons (5-10g)

1 Tablespoon (12g)

1-2 Tablespoons (12-24g)

More than 2 Tablespoons (>24g)

15. How many times in the past 6 months did you use vegetable margarine?

Never

Less than once a month

Once a month

2-3 times per month

Once a week

Twice a week

3-4 times per week

5-6 times per week

Once a day

Multiple times per day

16. Each time you used vegetable margarine, how much did you use?

Less than a Teaspoon (<5g)

1-2 Teaspoons (5-10g)

1 Tablespoon (14g)

1-2 Tablespoons (14-28g)

More than 2 Tablespoons (>28g)

17. Which margarine (brand and product) did you use?

Enter your answer

18. Where have you sourced your current information on Omega-3?

Internet/Social Media

Qualified Nutritionist/Dietician

Other Members of Sports Staff

Another Athlete

Family/Friend

Research Papers/Scientific Journals

19a. Do you currently or have you previously supplemented with an omega-3 fatty acid supplement or a fish oil supplement in the last six months? (i.e. omega-3 fish oil, cod liver oil, krill oil, algal oil)

Yes

No

19b. Please write down the name and brand of the products:

19c. How many times did you take the supplement or fish oil?

Less than once a month

Once a month

2-3 times per month

Once a week

Twice a week

3-4 times per week

5-6 times per week

Once a day

Multiple times per day

19.d. How much of the fish oil supplement did you consume? (e.g. 1 capsule, 2 sachet, 3 teaspoons)

20. If you used an omega-3 fatty acid supplement or a fish oil supplement (i.e. omega-3 fish oil, cod liver oil, krill oil, or algal oil) **in the previous 6 months, please indicate the reasons why.**

Enhance Recovery

Cardiovascular Health

Brain Function

Improve Body Composition

Reduce Inflammation

General Health

Prefer Not to Say

Don't Know

21. If you did not use an omega-3 fatty acid supplement or a fish oil supplement (i.e. omega-3 fish oil, cod liver oil, krill oil, or algal oil) **in the previous 6 months, please indicate the reasons why.**

Risk of Contaminants

Cost

Sufficient Intake from Diet

Ethical, Environmental or Lifestyle Reasons

Sensory Reasons (Taste, Smell, Aftertaste)

Medical Reason

Not Aware of Possible Benefits

22a. Have you consumed an omega-3 enriched products in the past 6 months that was not mentioned in the questionnaire? (i.e. enriched eggs or dairy, omega-3 recovery drink)

Yes

No

22b. If yes, please state which product you consumed and how often you consumed it?

If you have any comments about the questionnaire in general or about your diet in the last 6 months, you can write them down below
